# A Validated Volumetric Absorptive Microsampling-Liquid Chromatography Tandem Mass Spectrometry Method to Quantify Doxycycline Levels in Urine: An Application to Monitor the Malaria Chemoprophylaxis Compliance

**DOI:** 10.1155/2020/8868396

**Published:** 2020-12-15

**Authors:** Mylène Penot, Cyril Linard, Nicolas Taudon

**Affiliations:** Unité de Développements Analytiques et Bioanalyse, Institut de Recherche Biomédicale des Armées, 1 place du Général Valérie André, BP 73, Brétigny-sur-Orge 91220, France

## Abstract

Because of logistics and cost constraints, monitoring of the compliance to antimalarial chemoprophylaxis by the quantitation of drugs in biological samples is not a simple operation on the field. Indeed, analytical devices are fragile to transport and must be used in a perfectly controlled environment. This is also the case for reagents and supplies, and the waste management is constraining. Thus, samples should be repatriated. They should be frozen after collection and transported with no rupture in the cold chain. This is crucial to generate available and interpretable data but often without any difficulties. Hence, to propose an alternative solution easier to implement, a quantitation method of determining doxycycline in urine has been validated using a volumetric absorptive microsampling (VAMS^®^) device. As blotting paper, the device is dried after collection and transferred at room temperature, but contrarily to dried spot, the collection volume is perfectly repeatable. Analysis of VAMS^®^ was performed with a high-performance liquid chromatography coupled to a mass spectrometer. The chromatographic separation was achieved on a core-shell C18 column. The mean extraction recovery was 109% (mean RSD, 5.4%, *n* = 6) for doxycycline and 102% (mean RSD, 7.0%) for the internal standard. No matrix effect has been shown. Within-run as within-day precision and accuracy were, respectively, below 14% and ranged from 96 to 106%. The signal/concentration ratio was studied in the 0.25–50 *µ*g/mL range, and recoveries from back-calculated concentrations were in the 96–105% range (RSD < 11.0%). The RSD on slope was 10%. To achieve the validation, this new quantitation method was applied to real samples. In parallel, samples were analyzed directly after a simple dilution. No statistical difference was observed, confirming that the use of VAMS^®^ is an excellent alternative device to monitor the doxycycline compliance.

## 1. Introduction

Malaria is still a major public health problem affecting about half of the world population living in endemic areas each year. In 2017, about 219 million cases of malaria occurred worldwide and 435,000 people died, mostly children in the African region [1]. At the same time, 25 to 30 million people travel each year to endemic areas, leading to about 30,000 clinical cases [2]. Among nonimmune travelers, soldiers or workers are particular populations that may stay a few months under environmental conditions favorable for malaria transmission. Thus, compliance of prophylaxis rules is essential to preserve health and operational capabilities.

More than 10,000 French soldiers are deployed each year in malaria-endemic areas. Despite prophylactic countermeasures (vector protection, chemoprophylaxis (CP), health education, etc.), hundreds of malaria cases occur each year, inducing several unavailability days and sanitary evacuations within those; serious cases may lead to death [3–5]. For many years, CP in the French armed forces has mainly been ensured by a daily oral dose of 100 mg of doxycycline, which has been proved effective and well tolerated [6]. However, previous studies showed a strong correlation between malaria occurrence and lack of CP compliance [7]. If there is no doubt that combat setting may lead to this deficiency, it was also noted a premature shutdown of the CP taking medication (which should be extended four weeks for doxycycline) upon leaving endemic areas. It is interesting to note that this lack of CP compliance is not specific and has been described by other armies [8, 9] or civilian studies [10, 11].

Amongst CP monitoring tools, quantification of doxycycline in urine is an interesting approach. Indeed, doxycycline is eliminated in part through urine (above 35–60%) in its active form, allowing for noninvasive sampling [12]. Furthermore, urine concentration is about ten times higher than plasma concentration all that time [13], and maximal and minimal steady state plasma concentrations are within 0.5–4.0 *µ*g/mL range [14]. Thus, urinary doxycycline concentration is an observance marker several days after the last administration. Doxycycline is easily quantifiable with different techniques and in particular with (ultra-)high-performance chromatography liquid coupled to UV or mass spectrometry detector. However, with the lack of available ground tools, this quantitation is made in homeland in specialized laboratories. Taking doxycycline stability and the biological matrix handling into account, it is thus necessary to freeze samples at collection and to ship them under *ad hoc* conditions. This has a significant impact through both logistic and budgetary considerations. Indeed, freezing is rarely available in the field and the cost of sample transport in dry ice is expensive. To overcome these limitations, shipping of dried samples would be a good alternative. However, using the traditional collection on blotting paper is not available for a quantitative method due to poor reproducibility of the sampling.

Thus, this paper presents a quantitation method that has been fully validated using a perfectly calibrated microsampling device. After sample collection, the device can be sent to the homeland laboratory at ambient temperature in a simple postal parcel. Analysis is then performed on a liquid chromatography coupled to tandem-mass spectrometry system. The work here consisted in (i) the validation of a quantitation method responding to usual guidelines of the field [15–17], (ii) a stability study on the device, and (iii) analysis of samples from workers in malaria-endemic areas and those undergoing CP with doxycycline. In parallel, a direct quantification method after a simple sample dilution was validated and results obtained were compared with the microsampling method.

Results achieved in the presented work showed that doxycycline CP compliance in the field could easily be monitored using a fully calibrated volumetric absorptive microsampling (VAMS^®^) device. By extension, VAMS^®^ could be an interesting alternative to monitor large-scale populations [18, 19] and in particular to simplify the sample management during the preanalytical phase (transport and storage particularly).

## 2. Materials and Methods

### 2.1. Chemicals and Reagents

Doxycycline hyclate (HPLC purity ≥ 98%), tetracycline hydrochloride (HPLC purity ≥ 95%), and trifluoroacetic acid (TFA) were purchased from Sigma-Aldrich (St. Louis, MO, USA). Acetonitrile and methanol HiPerSolv CHROMANORM® (LC-MS grades) and acetic acid were obtained from VWR (Fontenay-sous-Bois, France). Ultra-high-quality water was obtained using a Milli-Q Integral 3 water-purifying system (Millipore, Bedford, MA, USA). *Mitra*® microsampling devices (10 *µ*L) were supplied by Neoteryx (Torrance, CA, USA). Urine samples used for the development and validation of the procedure were collected from healthy volunteers not undergoing drug therapy. These samples were aliquoted, kept frozen at −80°C, and then used during the study for the preparation of standard and quality control (QC) samples.

### 2.2. Instrumentation and Chromatographic Conditions

Optimization of various experimental parameters including the nature of the stationary phase, composition of the eluent, nature of the organic modiﬁer, ionization, and collision parameters was carried out (data not shown).

The chromatographic analysis was carried out using an Agilent 1100 chromatograph (Agilent Technologies, Les Ulis, France) equipped with a quaternary pumping unit, a degasser and an autosampler set at 6°C. Chromatographic separation was achieved using a Kinetex C18 100 Ǻ column (50 × 2.1 mm, 2.6 *µ*m) protected by a Kinetex C18 guard column (2.1 mm ID) purchased from Phenomenex (Le Pecq, France). The column temperature was set at 40°C, and the injected volume was 5 *µ*L. Chromatography was carried out *via* a gradient system at a ﬂow rate of 600 *µ*L/min. The mobile phase involved a mixture of eluent A, water-TFA (99.9 : 0.1, v/v), and eluent B, acetonitrile-acetic acid (99.5 : 0.5, v/v). The starting eluent mix of eluent A-eluent B was 90 : 10 (v/v), respectively; after 2 min the proportion of eluent B was increased linearly to 50% in 3.5 min and then increased linearly to 95% in 1 min, held for 1 min to wash the column, and then returned to its initial conditions within 1 min and re-equilibrated for 4 min.

The mass spectrometer was a 4000 QTRAP system (AB SCIEX, Les Ulis, France) equipped with an electrospray source that was run in positive mode (ESI+). Mass spectrometric data were acquired in multiple reactions monitoring mode. Nitrogen was used as the nebulizer, curtain, heater, and collision gas; associated settings were nebulizer gas (GS1) at 40 psi, heater gas (GS2) at 45 psi, and curtain gas (CUR) at 20 psi. The ion source was heated to 600°C, and the ion-spray voltage was set at 2000 V. The dwell time was fixed at 80 ms for each ion transition; fragments were formed under high-collision-activated dissociation gas (12 V). The parameters for doxycycline and tetracycline detection are described in Table 1.

During the method validation, two reference standard solutions prepared in a water-acetonitrile-TFA-acetic acid mixture (85 : 15 : 0.1 : 0.5, v/v) containing doxycycline at the concentration of low and high QC samples and the internal standard (IS) (1.0 *µ*g/mL) were injected into the LC-MS/MS system before each analytical run. This helps us to verify the performance of the system and to set up a suitability test procedure for routine application of the method by the calculation of many parameters as retention time for analyte and IS, signal/noise ratio, asymmetry factor, resolution, and the theoretical plate number.

### 2.3. Working Standard Solutions, Preparation of Calibration Curves, and Quality Control Samples

Individual stock solutions of doxycycline (2.5 mg/mL) and tetracycline (1.0 mg/mL) were prepared in the mixture of water-methanol (85 : 15, v/v), then aliquoted, and stored at −80°C. During the procedure validation, stock solutions were conserved at 4°C one week and were brought to room temperature before use (see Section 3.4). For each compound, two separate stock solutions were prepared: one was used for the preparation of calibrators and the other for the preparation of quality control (QC) samples. For doxycycline, 12 standard working solutions (concentrations ranging from 5.0 to 1.0 *µ*g/mL) were obtained extemporaneously by making appropriate dilutions of the stock solutions with the water-methanol mixture (85 : 15, v/v). The stock solution of the internal standard tetracycline was diluted 20-fold in water to reach a concentration of 50 *µ*g/mL.

The nominal concentration of calibration standards and QC samples were prepared by adding 10 *µ*L of working solutions to 190 *µ*L of drug-free matrix. Calibration curves consisted of 8 calibration points covering the 0.25–50.0 *µ*g/mL range, and they also included a blank matrix. Four levels of QC samples were prepared at the concentrations of 0.25 (lower limit of quantification QC samples), 0.30 (low QC samples), 20.0 (medium QC samples), and 35.0 *µ*g/mL (high QC samples).

### 2.4. Sample Preparation Procedure

Two sample preparation procedures were compared. The first one consisted in a simple dilution of the sample (50-fold) by the addition of 10 *µ*L of the internal standard solution (50.0 *µ*g/mL) and 480 *µ*L of the water-acetonitrile-TFA-acetic acid mixture (85 : 15 : 0.1 : 0.5, v/v/v/v) to 10 *µ*L of the sample. Analysis was performed after centrifugation at 25.000 × *g* for 10 min. The second procedure consisted in the absorption of 10 *µ*L of the sample on the microsampling device and a 2.5-h drying step at room temperature and protected from light; the time of contact between the device and sample was about 5 seconds. After the drying step, the adsorptive phase of the device was extracted in a 1.5 mL microtube with 500 *µ*L of the mixture of water-acetonitrile-TFA-acetic acid (85 : 15 : 0.1 : 0.5, v/v/v/v) containing the internal standard solution (1.0 *µ*g/mL). The tube was vortex-mixed during 30 min at room temperature and protected from light and then centrifuged (25,000 × *g*) for 10 min before analysis.

### 2.5. Data Analysis

Analyte peak areas were normalized to those of the internal standard and plotted versus concentration. To deﬁne the relationship between peak area ratios and nominal doxycycline concentrations in the matrix, two different models were tested: unweighted or weighted (i) linear regression model (*Y* = a*X* + *b*) and (ii) quadratic regression model (*Y* = a*X*^2^ + b*X* + *c*) in which *Y* is the peak area ratio and *X* is the nominal concentration of the analyte. The regression curve was not forced through zero. The resulting equation parameters were used to determine “back-calculated” concentrations. The good agreement between added and back-calculated concentrations of the calibrators was statistically evaluated. The normal distribution of the residuals (the difference between nominal and back-calculated concentrations) was verified. Moreover, the mean residual values (or mean predictor error) was computed and compared to zero (Student's *t*-test); the 95% conﬁdence interval was also determined.

### 2.6. Validation Procedure

The selectivity of the analytical methods was determined by the analysis of six different individual sources of the same biological matrix with and without spiking analytes and internal standards. The retention times of endogenous compounds in the matrices were compared with those of the compounds of interest for the evaluation of interferences; absence of interfering components was concluded for a signal in the free matrix below 20% and 5%, respectively, for the analyte at the lower limit of quantification and for the internal standard.

Linearity of the selected method was evaluated in the calibration range chosen for analysis with six calibration curves, validated by QC samples, made of eight calibration concentration levels, blank samples, and zero samples (processed matrix with IS)—model described in Section 3.5.

The lowest limit of quantification (LLOQ) was defined as the lowest concentration that could be determined with accuracy within 80–120% and a precision ≤20% on a day-to-day basis. To determine the analytical error for the LLOQ, the lowest standard of each calibration curve (*n* = 6) was used. The limit of detection was evaluated applying the formula (*a* + 3*σ*_a_)/b, where a and *σ*_a_ correspond to the average and the standard deviation of the intercepts and *b* corresponds to the average of the slope for the six calibration curves.

We assessed within-run precision and accuracy by analyzing six QC samples at each of the aforementioned four concentrations against calibration curves. We also performed the between-run precision and accuracy study: each of the four QC samples was analyzed twice a day, on six different days. The accuracy was evaluated as (mean calculated concentration/nominal concentration) × 100. Precision was given by the percent relative standard deviation (RSD).

For the microsampling procedure, extraction recoveries were measured six times at the concentration of low, medium, and high QC samples (*n* = 6 per concentration and per matrix batch). The areas under the peaks of extracted QC samples were compared with those of the samples that were spiked after extraction to characterize the absolute extraction recovery.

Carry-over was investigated at analyte and IS retention times in blank samples injected after the upper limit of quantification (ULOQ) for each calibration curve (*n* = 6).

Following guidelines, occurrence of matrix effects was investigated. Six individually different batches of doxycycline-free matrix from six healthy volunteers were treated as described above. Assay was performed at the concentration of low, medium, and high QC (*n* = 3 per concentration and per matrix batch).

For the simple dilution procedure, the analyte-to-internal standard peak area ratio was compared with the corresponding reference standard solution. For the microsampling procedure, working solution was substituted by the water-methanol mixture (85 : 15, v/v) on the microsampling device before the absorption step and then extracted with the mixture containing the analytes.

The matrix effect was calculated from the peak area ratios obtained from the reconstituted extracts (in presence of matrix ions), divided by the corresponding peak area ratios produced by the reference solutions. Matrix effect values between 85% and 115% were judged acceptable when relative standard deviation was inferior to 15%.

Evaluation of stability of stock and working solutions of the analyte and IS was carried out under conservation (−80°C, 4°C) and working (on bench) conditions. In the same way, evaluation of stability of samples was carried out under conservation (−80°C, −20°C) and working (on bench, 4°C, freeze and thaw) conditions. Finally, stability of the processed samples at room temperature (on bench) and 6°C (on autosampler) was studied. Totality of stability tests were done at two concentration levels of QC samples: low and medium, with three replicates for each.

### 2.7. Stability of Doxycycline on the Microsampling Device

The stability study of doxycycline in urine was performed by analyzing QC samples at low and medium concentrations (*n* = 3 per concentration) absorbed on the device against QC samples prepared extemporaneously in the above-mentioned conditions. After the drying step, the device was put into an opaque bag with a packet of desiccant agent. The bag was closed and stored at room temperature or −80°C. The duration of the study was 12 months.

### 2.8. Analysis of Urine Samples from Workers in Endemic Areas

The two different preparation methods were used to quantify doxycycline in urine samples from workers in malaria-endemic area and undergoing CP with 100 mg of doxycycline per day in one *per os* administration. Liquid samples were received frozen; after they were thawed at room temperature, absorption on the microsampling device was performed *versus* simple dilution, in order to compare the performance of the two sample treatment procedures with the objective to validate or not the potential gain of the microsampling device.

## 3. Results

### 3.1. Matrix Effect and Selectivity

**Figure 1:**
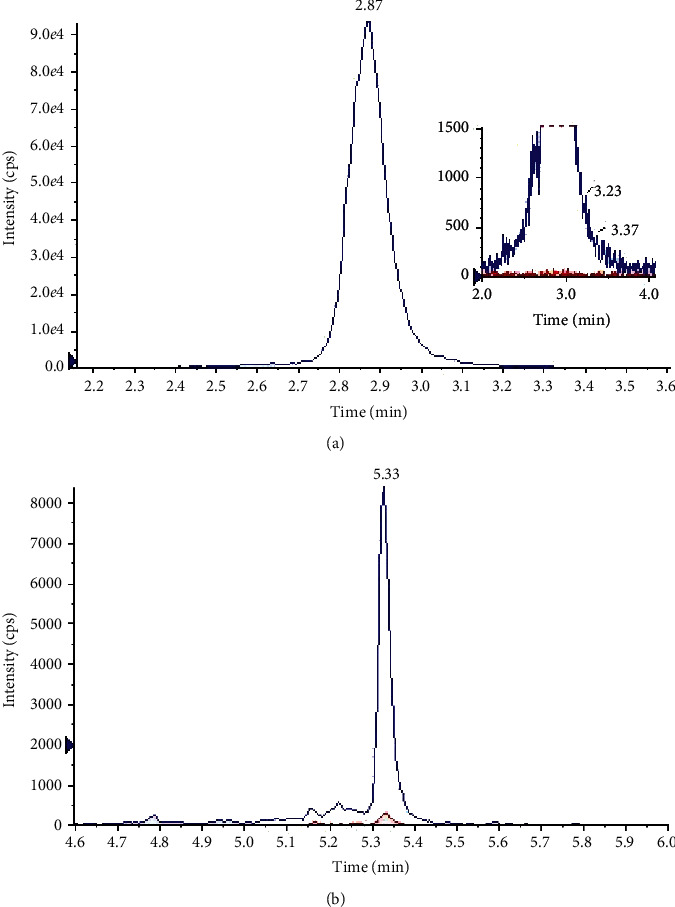
Overlay of typical multiple reactions monitoring mode chromatograms for tetracycline (a) and doxycycline (b) obtained from blank human urine (red line) and urine spiked with the LLOQ concentration (blue line) after extraction of the microsampling device.

Figure 1 shows typical chromatograms obtained from extracts of free urine, spiked or not with the analyte at the LLOQ. The selectivity of the two methods was demonstrated by representative chromatograms of blank matrices, which indicated that each peak of interest was well resolved from the matrix endogenous compounds; no interference was found. In addition, no tetracycline interference at the retention time of doxycycline signal was observed. Peak area ratios (reconstituted extracts/reference solution) of doxycycline were 1.19 and 1.12, respectively, with microsampling device and simple dilution (RSD, 11.5–17.7%). No statistical difference was observed between the three concentrations of doxycycline and the methods. Even through a slight matrix effect (average 109% and RSD 11%, on the three concentrations), the RSD of the ratio of doxycycline/IS per level of CQ were lower than 15%. The results were judged acceptable.

### 3.2. Drug/Response Relationship

Interday assays were determined for calibration curves prepared on different days (*n* = 6). For the two methods, quadratic calibration curves weighted by the concentration gave the best fit based on the statistical analysis results. The coefficient of determination was always higher than 0.996. The RSD values on the slope were 10.1% and 15.1% for microsampling device and dilution, respectively. For each point of the calibration curves, the concentrations were back-calculated from the corresponding quadratic equation parameters, and mean ± SD were calculated. Results are presented in Table 2. For concentrations obtained from calibration curves, the RSD around the mean value did not exceed 11.0%. The goodness of the fit between back-calculated concentrations and nominal concentrations was statistically verified: after simple linear regression, the slopes and intercepts were not statistically different from 1 to 0, respectively. Distributions of residuals were zero-centered and not correlated with concentration. In addition, the *t-test* showed that the bias was not statistically different from zero.

### 3.3. Accuracy, Precision, Extraction, Recovery, Carry-Over, Lower Limits of Quantification, and Detection

Precision was below 14%, and accuracy ranged from 96 to 106%. Individual results are presented in Table 3. These data are in correlation with the required validation criteria limits of the field.

The absolute mean recovery for the microsampling method, determined with six replicates for each QC level, was 108.7% (mean RSD, 5.4%). It was not statistically different over the range of the concentrations studied. For the internal standard, mean recovery was 101.9% (mean RSD, 7.0%).

Most of results are slightly above 100%, and it can be explained by a total extraction coupled to a concentration effect due to low solvent loss by absorption on the device, which was not taken into consideration in this calculation.

It was shown for the two methods that the carry-over is under control and complies with acceptance criteria of the field.

The targeted lower limit of quantification was 250.0 ng/mL. At this level, the signal/noise ratio on six different individuals matrices were measured in the range [7.3; 23.6] and [12.3; 37.7], respectively, with the microsampling device and simple dilution, and the precision was under 11.0% with an adequate accuracy.

Lower limit of detection was evaluated at 166 ng/mL.

### 3.4. Stability

Stability of stock solutions was proved for up to 5 days at 4°C and 12 months at −80°C, and working solutions were stable for 6 hours at room temperature.

In reconstituted extracts (i.e., in the autosampler at 4°C awaiting analysis), no significant losses occurred after 72 h, for both simple dilution and microsampling device extraction. Precision and accuracy were in the ranges of [0.5; 6.9%] and [91.6; 97.8%], respectively.

After storage away from light and humidity during 1, 3, 6, 9, and 12 months, and by comparison with extemporaneously prepared references, doxycycline was observed to be stable 1 month at room temperature for the two studied concentrations. Accuracy and precision were in the ranges of [91.0; 114.6%] and [2.5; 13.1%], respectively. After the first month, a loss in stability was observed. At 3 month, this loss of drug was above 50% and 20% for low and medium concentrations, respectively. After the 12-month period, this degradation was above 80% and 50%. However, no loss of drug was observed for the two concentrations after 12 months at −80°C. On the whole period, and for the two concentrations, the mean recovery was 101.7% (RSD, 6.8%).

### 3.5. Study on Samples from Volunteers Undergoing CP

**Figure 2:**
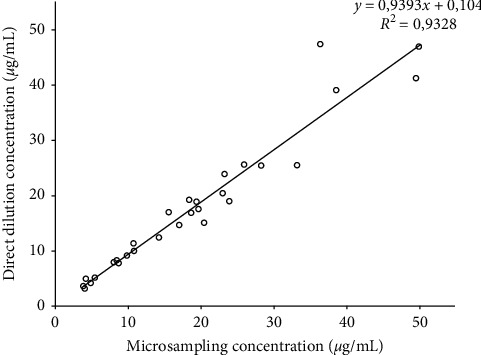
Concentrations of doxycycline in urine samples from workers under chemoprophylaxis quantified after direct dilution versus microsampling collection.

Figure 2 shows the quantification values obtained from twenty-nine urinary samples of volunteers taking one oral dose of doxycycline per day. Results for the two analytical methods were statistically the same: the straight line represents the concentrations of one method in relation to the other one, the coefficient of determination is 0.933, the slope is not statistically different from 1, and the intercept is not statistically different from 0.

Afterward, the microsampling device method was performed to quantify almost 100 samples. No results were under the LLOQ. The lowest values were greater than 1.0 *µ*g/mL, which is around the minimal expected value 24 h after the last administration, demonstrating that (i) all the volunteers were compliant when the sampling was performed and (ii) the method is fully suitable to serve this purpose.

## 4. Discussion and Conclusion

In the present paper, two sample treatment methods for the quantitation of doxycycline in urine, one by simple dilution and the other using a perfectly calibrated microsampling device, have been validated according to the most common bioanalysis guidelines.

In the context of malaria CP in adults, lowest expected concentrations 24 hours after the last daily doxycycline oral medication are in the 5–10 *µ*g/mL range. With a lower limit of quantification of 250.0 ng/mL and for doxycycline's plasma half-life above 16 h, a last medication dating four days back can be monitored. The stability of doxycycline on the device protected from light and humidity has been proved for 31 days at room temperature and 12 months at −80°C.

Results obtained on samples collected from workers under doxycycline CP were similar to the two methods, showing that the VAMS^®^ device is a relevant collection alternative. Furthermore, benefits of the VAMS^®^ device, in particular with no freezing and the shipping of samples at ambient temperature, are particularly evident. Easy to use, this microsampling device could be routinely employed and extended to other applications in the field. Nevertheless, it will still be necessary to check that shipping conditions from deployment areas do not modify the stability of drugs on the device.

## Figures and Tables

**Table 1:** Detection parameters of doxycycline and tetracycline.

Compound	Precursor (Da)	Dwell time (ms)	DP (V)	EP (V)	Quantifier			Qualifier		
					Product ion (Da)	CE (V)	CXP (V)	Production (Da)	CE (V)	CXP (V)
Doxycycline	[*M*+*H*] + 445.3	80.0	76	7	428.1	23	10	410.1	33	9
Tetracycline			57	11	427.1	20	5	410.1	25	9

**Table 2:** Relative standard deviation and recovery from back-calculated concentrations from calibration curves after microsampling and direct dilution (*n* *=* *6*).

Theoretical concentration (*µ*g/mL)	R.S.D (%)		Recovery (%)	
	Microsampling	Direct dilution	Microsampling	Direct dilution
0.25	10.2	8.1	103.7	100.6
0.40	7.5	6.8	98.3	99.8
1.00	4.1	8.4	97.8	99.6
5.00	11.0	10.2	100.0	98.5
12.5	6.0	4.2	98.3	102.5
25.0	5.1	3.8	105.0	99.4
37.5	4.8	2.5	95.9	99.2
50.0	3.0	0.6	101.0	100.5

**Table 3:** Precision and accuracy of the microsampling and direct dilution methods (*n* = 12).

Theoretical concentration (*µ*g/mL)	Precision (%)		Accuracy (%)	
	Microsampling	Direct dilution	Microsampling	Direct dilution

0.25	9.6	13.3	106.2	95.4
0.30	7.9	9.4	102.0	97.3
20.0	4.7	7.5	104.8	104.8
35.0	6.6	8.2	104.4	98.9

## Data Availability

All data used to support the findings of this study will be made available from the corresponding author upon reasonable request.
